# Wait and watch: A trachoma surveillance strategy from Amhara region, Ethiopia

**DOI:** 10.1371/journal.pntd.0011986

**Published:** 2024-02-22

**Authors:** Eshetu Sata, Fikre Seife, Zebene Ayele, Sarah A. Murray, Karana Wickens, Phong Le, Mulat Zerihun, Berhanu Melak, Ambahun Chernet, Kimberly A. Jensen, Demelash Gessese, Taye Zeru, Adisu Abebe Dawed, Hiwot Debebe, Zerihun Tadesse, E. Kelly Callahan, Diana L. Martin, Scott D. Nash

**Affiliations:** 1 Trachoma Control Program, The Carter Center, Addis Ababa, Ethiopia; 2 Disease Prevention and Control Directorate, Ministry of Health, Addis Ababa, Ethiopia; 3 Trachoma Control Program, The Carter Center, Atlanta, Georgia, United States of America; 4 Division of Parasitic Diseases and Malaria, Centers for Disease Control and Prevention, Atlanta, Georgia, United States of America; 5 Internships and Fellowships, Oak Ridge Institute for Science and Education, Oak Ridge, Tennessee, United States of America; 6 Research and Technology Transfer Directorate, Amhara Public Health Institute, Bahir Dar, Ethiopia; 7 Department of Health Promotion and Disease Prevention, Amhara Regional Health Bureau, Bahir Dar, Ethiopia; RTI International, UNITED REPUBLIC OF TANZANIA

## Abstract

**Background:**

Trachoma recrudescence after elimination as a public health problem has been reached is a concern for control programs globally. Programs typically conduct district-level trachoma surveillance surveys (TSS) ≥ 2 years after the elimination threshold is achieved to determine whether the prevalence of trachomatous inflammation-follicular (TF) among children ages 1 to 9 years remains <5%. Many TSS are resulting in a TF prevalence ≥5%. Once a district returns to TF ≥5%, a program typically restarts costly mass drug administration (MDA) campaigns and surveys at least twice, for impact and another TSS. In Amhara, Ethiopia, most TSS which result in a TF ≥5% have a prevalence close to 5%, making it difficult to determine whether the result is due to true recrudescence or to statistical variability. This study’s aim was to monitor recrudescence within Amhara by waiting to restart MDA within 2 districts with a TF prevalence ≥5% at TSS, Metema = 5.2% and Woreta Town = 5.1%. The districts were resurveyed 1 year later using traditional and alternative indicators, such as measures of infection and serology, a “wait and watch” approach.

**Methods/Principal findings:**

These post-surveillance surveys, conducted in 2021, were multi-stage cluster surveys whereby certified graders assessed trachoma signs. Children ages 1 to 9 years provided a dried blood spot and children ages 1 to 5 years provided a conjunctival swab. TF prevalence in Metema and Woreta Town were 3.6% (95% Confidence Interval [CI]:1.4–6.4) and 2.5% (95% CI:0.8–4.5) respectively. Infection prevalence was 1.2% in Woreta Town and 0% in Metema. Seroconversion rates to Pgp3 in Metema and Woreta Town were 0.4 (95% CI:0.2–0.7) seroconversions per 100 child-years and 0.9 (95% CI:0.6–1.5) respectively.

**Conclusions/Significance:**

Both study districts had a TF prevalence <5% with low levels of *Chlamydia trachomatis* infection and transmission, and thus MDA interventions are no longer warranted. The wait and watch approach represents a surveillance strategy which could lead to fewer MDA campaigns and surveys and thus cost savings with reduced antibiotic usage.

## Author summary

The return of trachoma transmission after elimination as a public health problem has been reached is a concern for control programs globally. Currently, many district-level trachoma surveillance surveys (conducted ≥2 years since elimination threshold has been reached) are resulting in a prevalence above the established threshold. Once a district returns above threshold, a program typically restarts costly mass drug administration campaigns and conducts more surveys. This study’s aim was to monitor recrudescence through a “wait and watch” approach within Amhara, Ethiopia. This entailed waiting to restart mass drug administration within 2 districts with trachoma prevalence above but close to the threshold at surveillance survey, then surveying the districts 1 year later using traditional and alternative indicators. These post-surveillance surveys assessed traditional trachoma signs, and additionally, children provided a dried blood spot for serology outcomes and a conjunctival swab for infection. The results of the study demonstrated that both districts had a prevalence below threshold with low levels of infection and serological evidence of transmission, and thus mass drug administration interventions are no longer warranted. The wait and watch approach represents a surveillance strategy that could lead to fewer surveys and mass drug administration campaigns and thus savings in programmatic costs with reduced usage of antibiotics.

## Introduction

Trachoma surveillance is conducted using population-based surveys designed to estimate the prevalence of trachomatous inflammation—follicular (TF) among children ages 1 to 9 years. TF is the programmatic indicator measured through field grading of the everted eyelid and serves as a proxy indicator for *Chlamydia trachomatis* infection [1]. Impact surveys are conducted after 1 to 5 years of surgery, antibiotics (i.e., mass drug administration [MDA] for affected populations), facial cleanliness, and environmental improvement (SAFE) strategy interventions. Once a district has a TF prevalence <5% at impact survey, it is recommended that trachoma programs stop MDA for a period of ≥2 years before conducting a trachoma surveillance survey (TSS). If a district sustains a prevalence of TF <5% at this TSS, antibiotic interventions are no longer required for the district. Once all districts in a trachoma-endemic country reach <5% TF, and maintain <5% at TSS, then that country has reached elimination as a public health problem for this indicator [2].

Recrudescence of trachoma after the elimination as a public health problem threshold has been reached is a serious concern for trachoma control programs globally. Currently, many district-level TSS show a TF prevalence above the elimination threshold (≥5%) [3]. This problem is particularly acute in Ethiopia, where >50% of TSS have returned results above threshold. Once a district returns to TF ≥5%, a program typically restarts costly MDA campaigns, and then must repeat impact surveys and TSS. In the Amhara region, Ethiopia, most TSS resulting in a TF prevalence ≥5% have a prevalence close to 5%, making it difficult to determine whether the result is due to true recrudescence or to statistical variability [4].

This study’s aim was to monitor recrudescence within Amhara using a “wait and watch” approach, designed to allow programs to make more informed decisions about whether additional rounds of MDA are required after an unfavorable (≥5% TF) TSS result. This approach included refraining from restarting MDA in 2 districts with a TF prevalence ≥5% at TSS and instead resurveying the districts 1 year later using traditional and alternative trachoma indicators, including measures of infection and serology. A 1-year follow up period was chosen to allow for more time for TF to resolve within the selected districts, assuming low levels of transmission, and to also allow for the program to catch any potential increase in transmission early should it occur. This approach may provide programs with a surveillance strategy that leads to reductions in programmatic costs and in unnecessary antibiotic distributions as well as reductions in timelines required to reach the elimination as a public health problem threshold.

## Methods

### Ethical review

The study protocol was approved by Emory University’s Institutional Review Board (IRB) under protocol number 079–2006. The Ministry of Health and the Ministry of Science and Technology of Ethiopia, the Amhara Regional Health Bureau, and Tropical Data (https://www.tropicaldata.org/) also reviewed and approved the protocol. Staff members of the Centers for Disease Control and Prevention (CDC) did not have contact with study participants or access to identifying information and were determined to not be engaged in research on human subjects. Because of the high illiteracy rate in the population, IRB approval was received for individual and parental oral consent or assent for older children. Oral consent or assent was recorded electronically for all individuals participating in the study according to the Declaration of Helsinki.

### Setting and historical results

**Fig 1:**
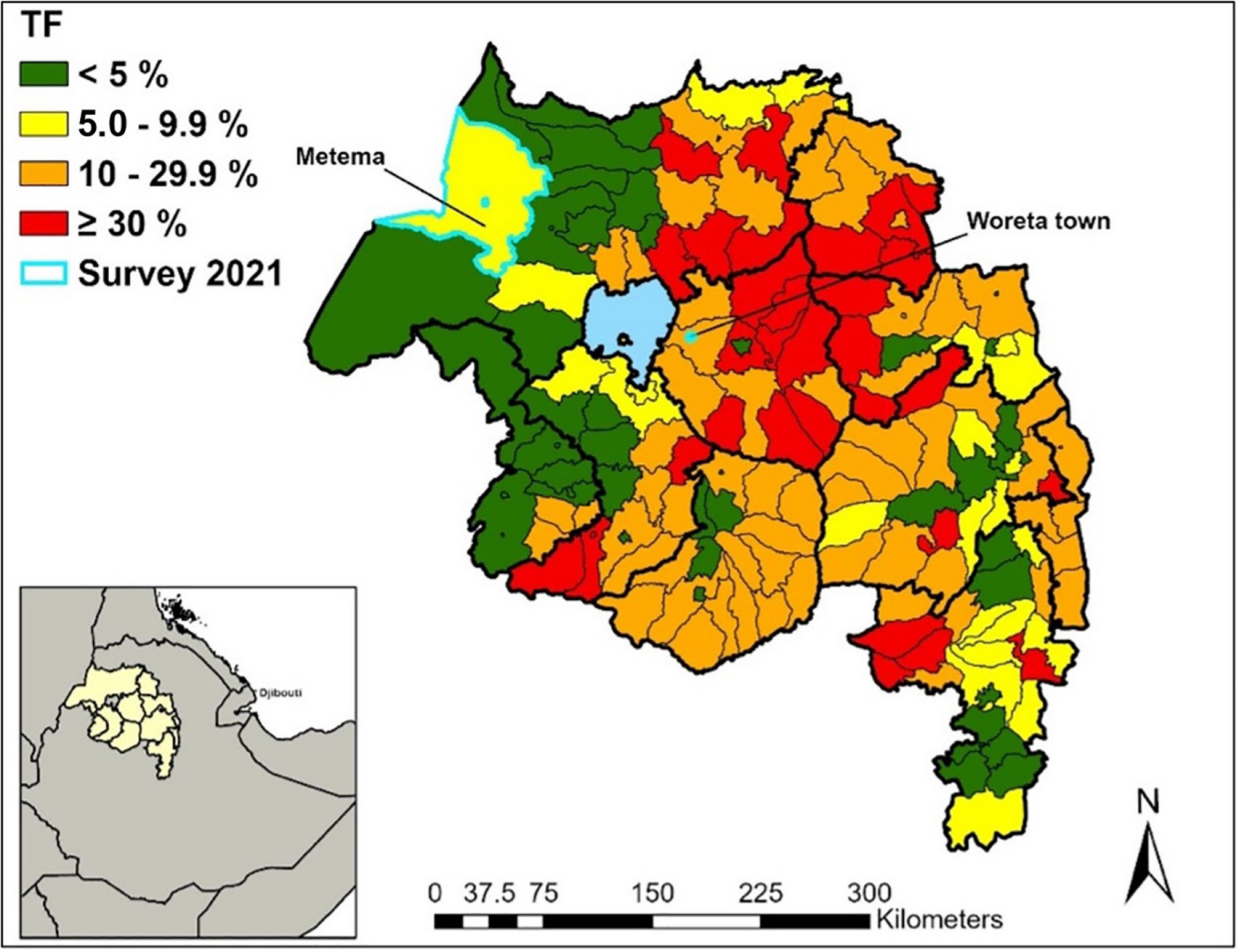
Selected districts for the wait and watch surveillance approach, Amhara, Ethiopia 2021. Map created in ArcGIS Pro 2.2.6 (ESRI, Redlands, CA) using a shapefile sourced from the GADM database (gadm.org).

Metema is a large rural district in the Northwest zone of North Gondar, and Woreta Town is a peri-urban district in the central zone of South Gondar (Fig 1). In 2017 both Metema and Woreta Town districts reached the <5% TF threshold for the first time. Prior to the 2017 survey, Woreta Town had been surveyed 3 times to measure impact and had received 7 years of SAFE interventions. Metema had been surveyed once previously and had received 9 years of SAFE interventions. During the 2019 TSS both Metema (5.2%) and Woreta Town (5.1%) had a TF prevalence just greater than the threshold. After consultation with the Amhara Regional Health Bureau and the Ministry of Health, these 2 districts were chosen because they were close to, but over 5% TF, and they represented two potentially different epidemiological settings. One, Metema, a rural district bordered by districts with low trachoma endemicity, and Woreta Town, a peri-urban district surrounded by a district with higher trachoma endemicity. The Trachoma Control Program in Amhara did not administer MDA in these districts in 2020; both districts were then surveyed in 2021. As of the 2021 surveys, neither district had received MDA for a period of approximately 4 years (2017–2020).

### Survey design

Wait and watch post-surveillance surveys were multi-stage cluster surveys whereby in the first stage, clusters (communities) were selected using a probability proportional to estimated size sampling method. In the second stage, 1 “segment” of approximately 30 households (called a development team in Ethiopia) was randomly chosen. All individuals ages ≥1 year residing in all households in each development team were eligible for the survey.

The sample size calculation for each survey assumed 4% TF ±2%, a design effect of 2.63, and a non-response inflation factor of 1.2; and thus 1,164 children ages 1 to 9 years were targeted for examination. Assuming children make up 27% of the population of Amhara, a total of 30 clusters of 30 households were sampled in each district to reach the desired sample size [4].

### Training

Grader trainings conducted in Amhara have been described previously [4,5]. Trainees were trained to identify the active trachoma signs TF and trachomatous inflammation—intense (TI) through classroom and field experiences. Graders who passed a slide-based inter-grader assessment (IGA) on identifying these signs went on to take a field-based IGA. In the field-based IGA, trainees graded the conjunctivae of 50 children. To pass the field IGA and be certified as a grader, trainees were required to receive a kappa of ≥0.7 when compared to a single certified grader trainer. Graders who had completed a training or participated in a survey in the last 12 months were also eligible to take part in the surveys.

Data collectors and technicians responsible for sample collection participated in a 3-day training comprised of classroom and field practice. Beyond data and sample collection, training covered consent procedures and cold chain maintenance.

### Data collection

After obtaining consent, survey teams conducted a household questionnaire to capture information on water, sanitation, and hygiene (WASH) variables. Graders were trained on observing faces for ocular and nasal discharge via slide presentations and community observations. Clean face was defined as absence of ocular and nasal discharge on the faces of children ages 1 to 9 years [4,5]. Household members present at the time of the visit were graded for the signs of trachoma. Using a 2.5x magnifying loupe and adequate light, graders assessed each conjunctiva for TF and TI according to the WHO simplified grading scheme [6]. Data were entered into the Tropical Data application on cellphones. Individuals identified as positive for TF and/or TI were offered tetracycline eye ointment.

Within all 30 clusters, all consenting, examined children ages 1 to 5 years were swabbed to test for ocular *C*. *trachomatis* infection. After the clinical examination, a gloved grader passed the conjunctival swab (Fisher Scientific, Waltham, MA) 3 times over the conjunctivae, rotating the shaft of the swab 120 degrees after each swipe [7]. The grader then placed the swab into a dry 2 mL vial being held by a gloved “tuber.” This tuber placed the cap on the vial, labeled the tube, and placed the tube into a box inside a cooler with ice packs. One child in each community was selected for a negative control “air swab,” whereby a swab was passed within 1 inch of the conjunctiva without making contact. This air swab was placed in an identical tube, labeled, and stored with the conjunctival swabs. Conjunctival samples were stored at -20°C for testing in the laboratory.

Trained technicians traveled with data collection teams, took finger sticks using a lancet, and collected approximately 60 μL of blood on filter paper (TropBio Pty Ltd., Townsville, Queensland) from children ages 1 to 9 years. Each filter paper was dried a minimum of 2 hours and then placed into a sealable plastic bag. Filter papers were stored in coolers until they were placed in -20°C freezers in the laboratory.

### Laboratory procedures

All conjunctival samples were assayed at the Amhara Trachoma Molecular Laboratory at the Amhara Public Health Institute in Bahir Dar, Ethiopia. Samples were first randomized within each district, and then pooled, 5 individual samples to 1 pool before assaying. Pools were tested for *C*. *trachomatis* DNA using the RealTime polymerase chain reaction (PCR) assay (Abbott Molecular, Des Plaines, Illinois) on the m2000 system. To generate individual results, samples which formed positive pools were run again individually. To obtain a measure of chlamydial load, a calibration curve was created prior to individual testing using a standard set of elementary body (EB) titrations [8]. Technicians were masked to whether a sample was from a participant or was an air swab and masked to the district or trachoma clinical status of the individual providing the swab. Laboratory quality control procedures have been published previously [7,9].

All dried blood spots (DBS) specimens were shipped at ambient temperatures to CDC in the U.S. where they were tested for antibodies to *C*. *trachomatis* antigens Pgp3 and CT694 on a multiplex bead assay. Antigen-coupled beads were added to 96-well plates (Millipore, Bedford, MA). Control sera and blood spot eluates (1:400) were then added to appropriate wells, beads suspended, plates covered, and shaken at room temperature for 1.5 hours. After washing the beads, total IgG was detected using biotinylated mouse anti-human total IgG (clone H2; Southern Biotech, Birmingham, AL) and biotinylated mouse anti-human IgG4 (clone HP6025; Invitrogen, South San Francisco, CA). After a second wash, streptavidin-phycoerythrin (SAPE, Invitrogen) was added at a concentration of 250 ng per well and incubated for 30 minutes at room temperature. Beads were read on a Luminex instrument (Luminex Corp., Austin, TX) equipped with Bio-Plex Manager 6.0 software (Bio-Rad, Hercules, CA). The signal was converted to median fluorescence intensity (MFI) with background levels subtracted out (MFI-BG). Positivity thresholds were generated using a receiver operating characteristic curve panel of specimens of previously classified positive or negative samples. The MFI-BG thresholds were 783 for Pgp3 and 102 for CT694.

### Statistical analysis

District-level prevalence estimates for TF were provided by the Tropical Data service and were calculated by taking the median age-adjusted (using the Ethiopian National Census population) cluster prevalence estimates from within each district [10]. Confidence intervals (CI) were calculated using a previously described bootstrapping method [10]. Prevalence estimates for TI, clean face, Pgp3, and CT694 were calculated in a similar manner to TF. CI for these and other WASH variables were also estimated in the same manner as those for TF. Logistic regression was used to test the associations between age and TF and was adjusted for clustering at the cluster and household levels using survey procedures in R. *C*. *trachomatis* infection prevalence was estimated from the pooled district prevalence as the number of positive individual samples most likely to have resulted in observed pooled results using maximum likelihood methods [7,11]. Antibody data were described as continuous and binary variables based on the above thresholds. *C*. *trachomatis* force of infection was also estimated by the seroconversion rate (SCR) per 100 children per year (100 child-years) to Pgp3 and CT694 among children ages 1 to 9 years from age-structured seroprevalence using a generalized linear model with a complementary log–log link and robust standard errors [12–14]. The model assumed a constant force of infection across the age span. Map created in ArcGIS Pro 2.2.6 (ESRI, Redlands, CA) using shapefiles sourced from the GADM database (gadm.org).

## Results

Between March and April 2021, 30 clusters were surveyed in each wait and watch district (Table 1). Among 1,585 children ages 1 to 9 years enumerated, 1,554 (98%) were examined for trachoma signs and 1,508 (95%) provided DBS. Among 1,008 children ages 1 to 5 years examined, conjunctival swabs were collected from 939 (93.2%). The prevalence of WASH indicators such as presence of household latrine and improved water source was greater than 90% in Woreta Town, while the prevalence of household latrine (32.8%) and improved water source (38.2%) was considerably lower in rural Metema (S1 Table).

**Table 1:** Sample sizes for wait and watch districts, Amhara, Ethiopia, 2021.

District	Estimated population	Clusters	Households	Children enumerated 1-9y	Clinical assessment 1-9y	DBS samples 1-9y	Children examined 1-5y	Ocular swabs 1-5y
Metema	158,198	30	900	936	918	879	547	510
Woreta Town	48,404	30	899	649	636	629	461	429
Total		60	1,799	1,585	1,554	1,508	1,008	939

**Fig 2:**
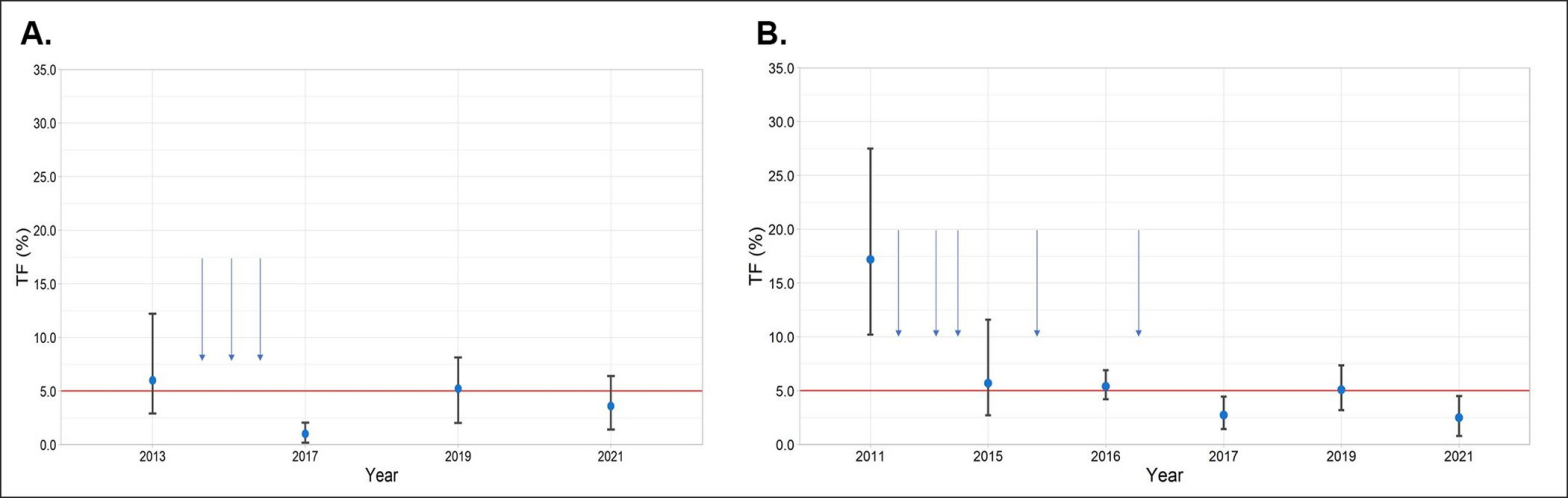
District prevalence of TF among children ages 1 to 9 years over time in A) Metema and B) Woreta Town. Horizonal arrows represent mass drug administration in Metema (2014, 2015, 2016) and in Woreta town (2011, 2013, 2014, 2015, 2016).

The TF prevalence among children ages 1 to 9 years in Metema was 3.6% (95% CI:1.4–6.4) and in Woreta Town was 2.5% (95% CI: 0.8–4.5) (Fig 2A and 2B). TF prevalence decreased with age among children in this age group (Metema: Ptrend = 0.016; Woreta Town: Ptrend = 0.002) (S1 Fig). The prevalence of TI in Metema was 0.2% (95% CI: 0.0–0.5%) and in Woreta Town was 0%.

**Fig 3:**
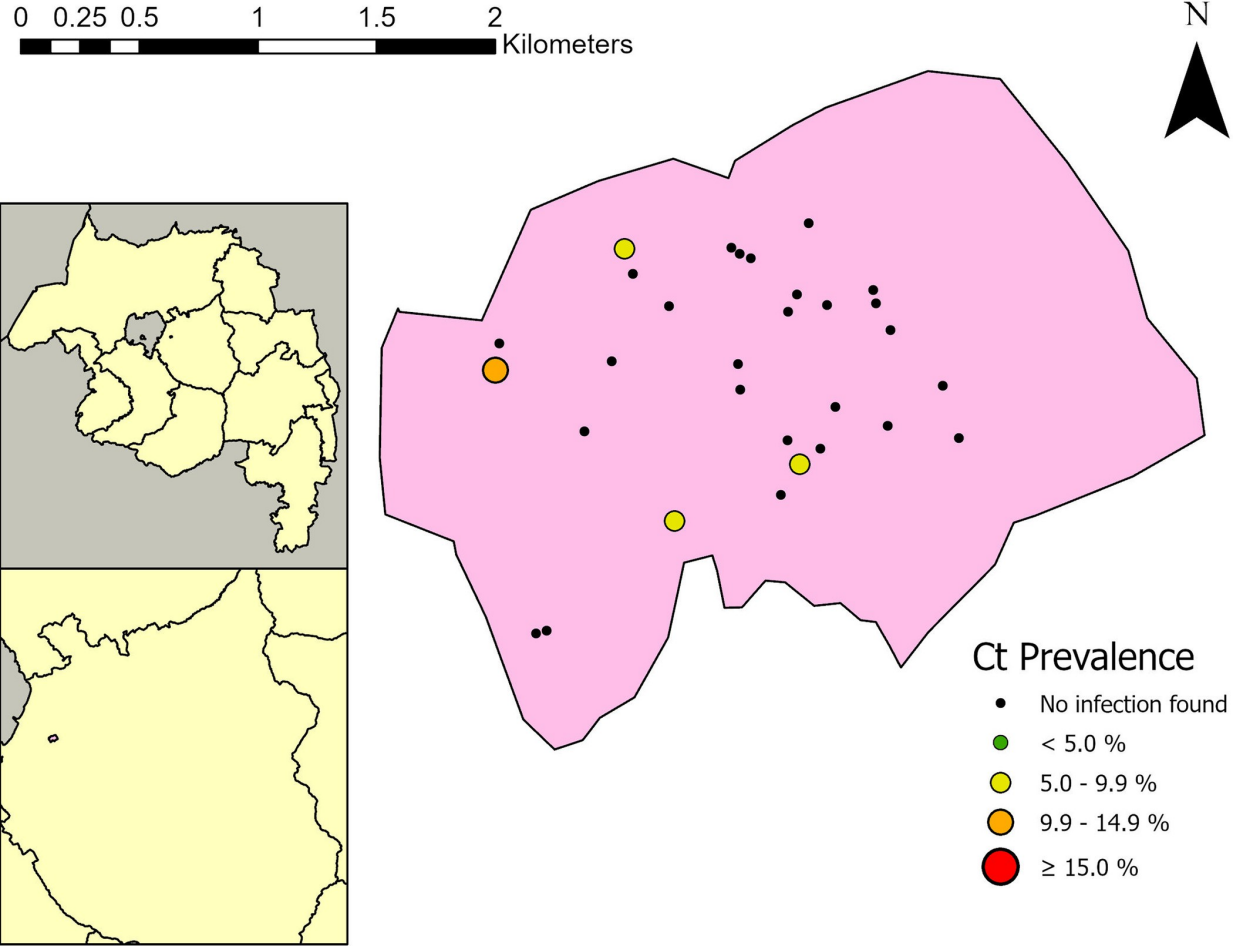
Cluster specific *Chlamydia trachomatis* (Ct) infection prevalence in Woreta Town district, Amhara, Ethiopia 2021. Map created in ArcGIS Pro 2.2.6 (ESRI, Redlands, CA) using a shapefile sourced from the GADM database (gadm.org).

*C*. *trachomatis* infection was detected only in Woreta Town, with a prevalence of 1.2% among children ages 1 to 5 years. This represented a total of 5 cases found in 4 communities within the district (Fig 3). Geographically, these communities were fairly spread out throughout the district. Among the 5 children with infection, the median EB load was 312.0 EB (Interquartile range: 52.3–1216.7 EBs). Among children with infection, 2 also had TF, 4 were seropositive to CT694 and all 5 were seropositive to Pgp3. These 5 infected children made up 5/11 (45.5%) of the children ages 1 to 5 years positive for Pgp3 and 5/44 (11.4%) of those positive for CT694 in Woreta Town. All 60 (100%) of air swabs collected in these two districts were negative.

**Fig 4:**
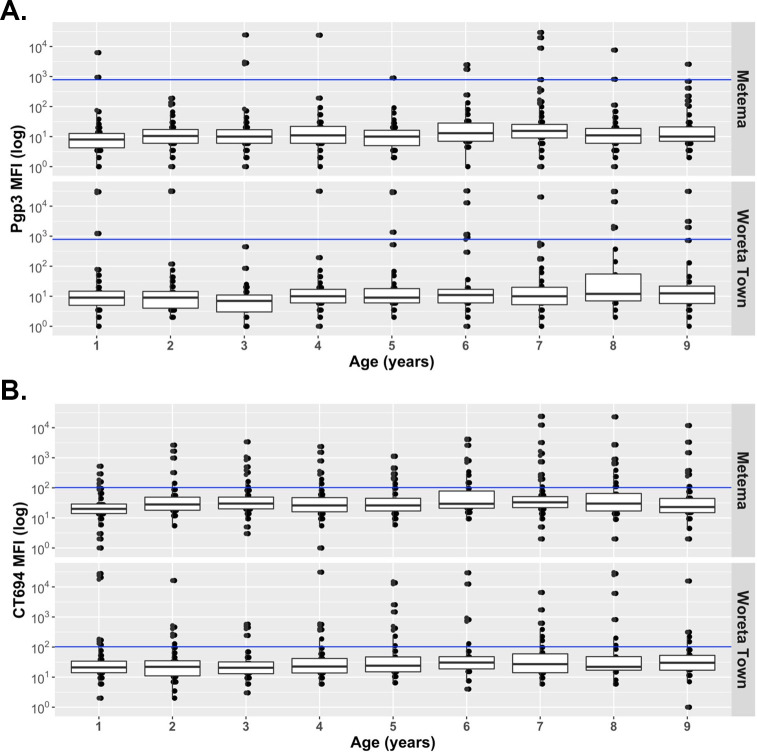
Median MFI Distribution of A) Pgp3 and B) CT694 among children ages 1 to 9 years in Metema and Woreta Town districts, Amhara, Ethiopia, 2021.

**Fig 5:**
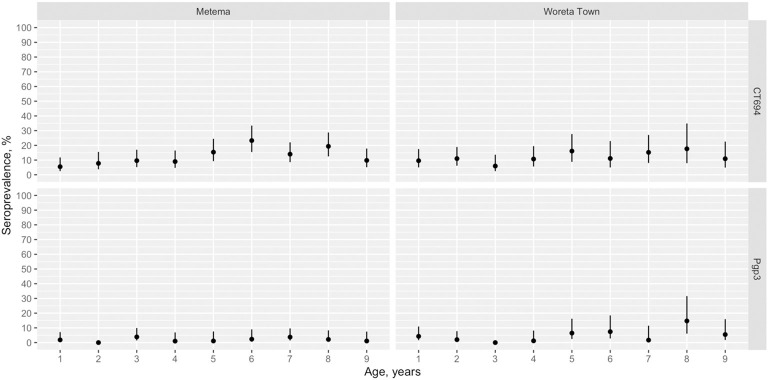
Age-seroprevalence among children ages 1 to 9 years in Metema and Woreta Town districts, Amhara, Ethiopia, 2021.

**Fig 6:**
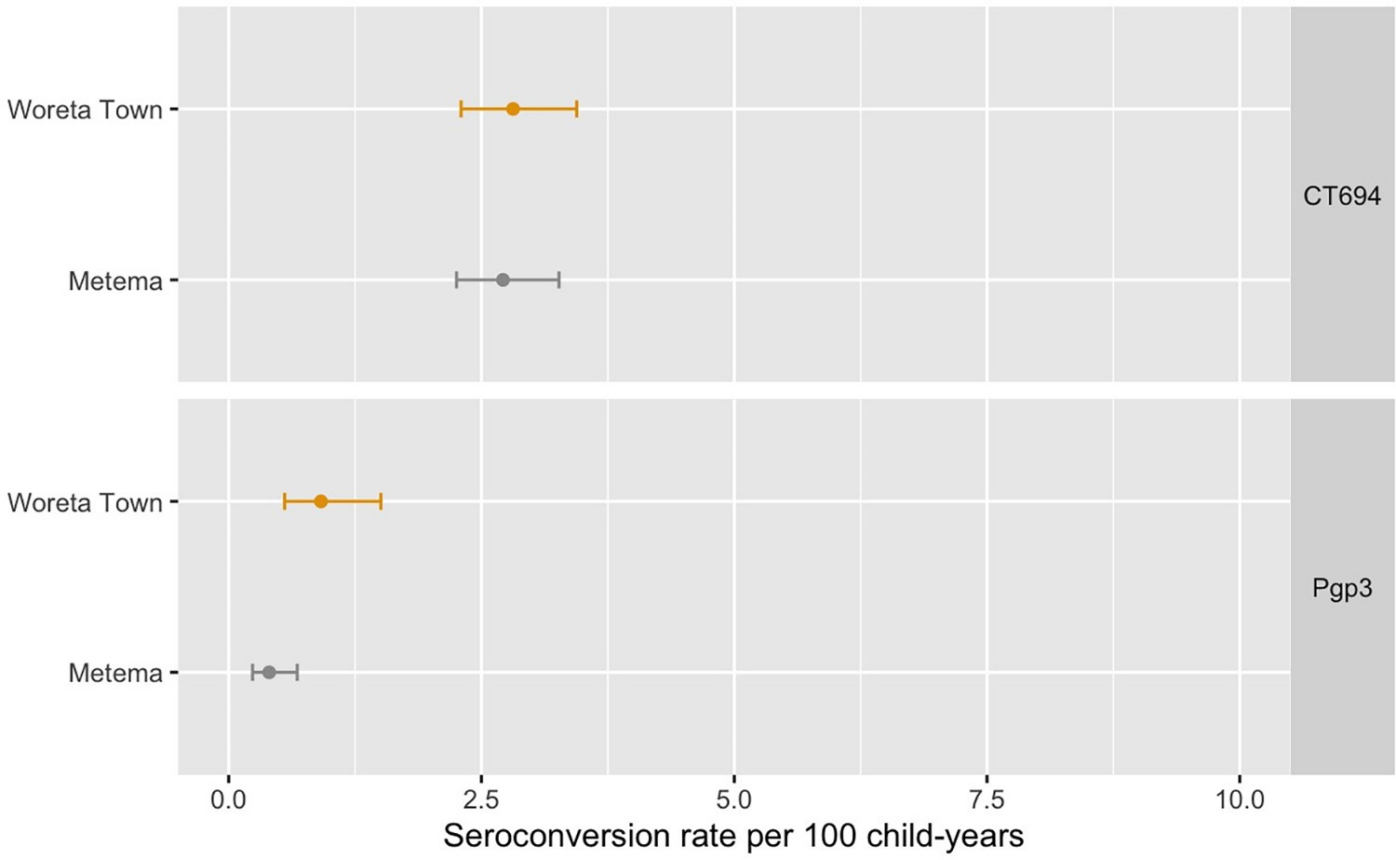
Seroconversion rates (SCR) per 100 child-years to Pgp3 and CT694 among children ages 1 to 9 years in Metema and Woreta Town districts, Amhara, Ethiopia, 2021.

In both Metema and Woreta Town, antibody levels to Pgp3 and CT694 were lower than the cutoff for seropositivity for most children ages 1 to 9 years. (Fig 4A and 4B). Among children in this age group in Metema, the seroprevalence to Pgp3 was 2.4% (95% CI: 1.4–3.5%) and to CT694 was 13.6% (95% CI: 11.1–16.2%) (Fig 5). In Woreta Town, the seroprevalence to Pgp3 and CT694 among this age group was 3.6% (95% CI: 2.0–5.4%) and 9.7% (95% CI: 7.7–11.8%), respectively. The SCR among children ages 1 to 9 years was 0.4 (95% CI: 0.2–0.7) seroconversions per 100 child-years for Pgp3 and 2.7 (95% CI: 2.3–3.3) for CT694 in Metema and 0.9 (95% CI: 0.6–1.5) for Pgp3 and 2.8 (95% CI: 2.3–3.4) for CT694 in Woreta Town (Fig 6).

## Discussion

Trachoma prevalence was below the elimination threshold in these 2 wait and watch districts, 4 years after the cessation of MDA. The results from the alternative indicators *C*. *trachomatis* infection and antibody responses to chlamydial antigens further demonstrated that transmission was low. Although these districts had a TSS result >5% 1 year prior to these post-surveillance surveys, which traditionally would have resulted in the re-enrollment in antibiotic interventions and subsequent surveys, these results demonstrated that true trachoma recrudescence was not observed. Given the evidence generated by these surveys, there is no need for further MDA in these districts. The wait and watch approach can provide useful information to programs to inform intervention decisions for districts near the elimination threshold.

Despite the generally high trachoma endemicity of Amhara, the region has observed successes in reducing the prevalence of TF to below the elimination as a public health problem threshold within individual districts. Surveillance surveys, first conducted in the region in 2015, demonstrated that districts could maintain a prevalence below threshold even when surrounded by neighboring endemic districts [15]. By 2019, 37 district-level TSS had been conducted, and 28 (76%) remained below threshold. Of those 9 districts which had unfavorable (>5% TF) TSS results, 6 had a TF prevalence between 5 and 10% and 3 had a TF prevalence >10%. Of the 6 districts with TF between 5 and 10%, 5 had a prevalence <6%. In a review of global trachoma data, it was also observed that most unfavorable TSS had a TF prevalence <10% [16]. While the trachoma community has begun using the term recrudescence to mean any district that has an unfavorable TSS result, it is clear from these data that a TF just above threshold may not mean the actual return of *C*. *trachomatis* transmission in a district [17]. A considerable proportion of these unfavorable results are likely due to statistical variability around the 5% threshold as demonstrated in recent modeling [18]. Given the cost of restarting MDA and the follow-up impact and TSS surveys that would be required, consideration of increased surveillance within districts close to the elimination threshold, with the inclusion of indicators better suited to measure disease transmission could be an alternate strategy.

These post-elimination surveys demonstrated that Woreta Town, a peri-urban district in the center of South Gondar zone, had a TF prevalence <5% 4 years after the last MDA. Further, the SCR to Pgp3 estimated in this 2021 study (0.9) was nearly identical to the SCR estimated in 2017 (1.0) when the TF prevalence was 2.7% [12]. Therefore, at a district level, it appears that *C*. *trachomatis* transmission has been consistently low despite an unfavorable TSS result in the intervening years. While Woreta Town is surrounded by a district with higher endemicity, infection may have remained low due to the high prevalence of water and hygiene indicators observed in this district. At this current survey, however, several of the children assessed were positive for *C*. *trachomatis* infection and were concurrently seropositive for Pgp3. This suggests that some low level of transmission, enough to result in seroconversion among young children, was still occurring in some areas of Woreta Town. In Amhara, detecting infection once a district is below the TF threshold is not uncommon (17/65, 26%; median infection prevalence = 1.5%), however, it is difficult to know the programmatic implications of a low level of infection [9]. At the time of these surveys, Woreta Town was surrounded by trachoma-endemic districts, and South Gondar zone has been historically one of the worst trachoma affected zones in the region [5,7,19,20]. Population mixing, between residents of Woreta Town and those from surrounding endemic districts, may be occurring. More research is needed to understand transmission dynamics within the urban and peri-urban districts served by trachoma control programs. Future operational research to determine the significance of low level, localized *C*. *trachomatis* transmission on district-wide trachoma recrudescence is needed.

The inclusion of alternative trachoma indicators, *C*. *trachomatis* infection and serology, generated a more complete picture of the trachoma transmission in these 2 settings. This study was made possible by the fact that this Program has monitored *C*. *trachomatis* infection since 2011 and serology since 2017 [7,12]. These indicators helped demonstrate that transmission was no longer a problem in these 2 districts. In both districts, CT694 responses were consistently higher than Pgp3 responses across the age range. Reasons to explain this finding could be that CT694 may be a longer-lived antibody or that it requires fewer infections for someone to seroconvert to CT694 compared to Pgp3. The consistently low Pgp3 seroprevalence appeared to be more reflective of the low level of infection and TF prevalence observed in these districts and others, suggesting that Pgp3 as a single measure of antibody response may be a reliable indicator [21]. As more countries collect serological and infection data within their programmatic surveys, the cumulative data may help understand the comparative usefulness of these 2 indicators to trachoma surveillance and may help in the development of actionable programmatic thresholds [21]. Because of the wait and watch approach, at least 200,000 doses of azithromycin were conserved in these 2 districts in 2020. Administering medications when they are not needed is not only costly for programs, but it may contribute to the possibility of antimicrobial resistance in off-target organisms such as *Streptococcus pneumoniae* and *Staphylococcus aureus* among populations [22].

The enrollment of only two districts into this study may have limited the generalizability of the findings; however, one of the districts was rural, and the other peri-urban, and both were found in areas with historically high levels of trachoma [5,23]. Although one district in this study was surrounded by highly trachoma endemic districts (Woreta Town) and one had low levels of WASH infrastructure (Metema), more operational research is needed as to how wait and watch surveillance may operate under varying geographic and environmental conditions. The wait period used in this study was approximately 1 year, however, longer wait times before the follow-up survey could be considered. The availability of additional indicator data, *C*. *trachomatis* infection and serology, at the initial TSS may aid in this decision. Further, these districts would make good candidates for post-endemic surveillance, returning in the future to better understand the long-term sustainability of remaining below the elimination threshold. In this study both districts had a TF prevalence just above the 5% threshold at TSS. Future programmatic research should consider assessing how the wait and watch strategy would work among districts with a higher TSS TF prevalence, closer to 10% for example. A current trial in Niger is testing a similar approach, randomly assigning districts with a TF between 5 and 20% to three additional years of MDA or to ceasing MDA all together [24].

While the inclusion of infection and serological monitoring was useful to understand transmission in these settings, and thus helped lead to better programmatic decision making, these methods come with a cost above and beyond standard trachoma surveys [25]. As programs move towards enhanced surveys with the inclusion of infection or serology measures, there needs to be an understanding of the costs and feasibility of including such measures. Work is ongoing to understand the additive costs of enhanced trachoma monitoring in Amhara. The decision to choose one or both enhanced indicators should further be driven by available capacity within the country to ensure high quality laboratory testing [26]. The development of a lateral flow assay for serology may increase the feasibility of this indicator for programmatic use [27]. Alternate considerations could include moving forward with the wait and watch approach, monitoring the clinical signs of trachoma, but without the infection and serological components. Recent guidance from a WHO meeting of global experts has recommended a similar approach as an option for programs to consider [17].

## Conclusion

The wait and watch study in Amhara demonstrated that an unfavorable result at TSS, a TF ≥5% but close to the threshold, may not mean true recrudescence of trachoma transmission. Within the 2 study districts, TF returned below threshold 1 year later, and accompanying alternative trachoma indicators evidenced low levels of transmission. The wait and watch approach represents a surveillance strategy for trachoma control programs which could reduce the human and financial resource demand on programs required for MDA and surveys and limit unnecessary antibiotic treatments over the long term.

## Supporting information

S1 Table District prevalence (95% confidence intervals) of key water, sanitation, and hygiene (WASH) indicators, Metema and Woreta Town districts, Amhara, Ethiopia, 2021. (DOCX)

S1 Fig Age-specific prevalence of TF among children ages 1 to 9 years, Amhara, Ethiopia, 2021. (DOCX)
