# Supplementary material for: Wait and watch: A trachoma surveillance strategy from Amhara region, Ethiopia
Source: PLoS Negl Trop Dis. 2024 Feb 22;18(2):e0011986. doi: 10.1371/journal.pntd.0011986 (PMC10914254; doi:10.1371/journal.pntd.0011986)
Supplement: S1 Table — (DOCX) [file pntd.0011986.s001.docx]

Supplemental Table 1. District prevalence (95% confidence intervals) of key water, sanitation, and hygiene (WASH) indicators, Metema and Woreta Town districts, Amhara, Ethiopia, 2021.

| District | Water within 30 minutes | Access to latrine | Access to improved latrine | Access to improved water source | Clean face observed among children ages 1 to 9 years |
| --- | --- | --- | --- | --- | --- |
| Metema | 42.6%  (30.9, 54.9) | 32.8%  (21.8, 44.5) | 7.3%  (2.9, 12.5) | 38.2%  (22.6, 54.4) | 69.2%  (61.7, 76.6) |
| Woreta Town | 96.0%  (88.9, 100) | 95.9%  (92.0 98.9) | 49.2%  (41.0, 58.1) | 100% | 62.5%  (56.0, 68.8) |

Improved latrine = pit latrine with slab or pour/flush toilet; Improved water source = protected spring, hand pump/borehole, piped water, or rainwater collection; Water within 30 minutes = time to access bathing or drinking water and return home. Clean face was defined as absence of ocular and nasal discharge on the faces of children ages 1 to 9 years.
