# Supplementary figures and images for: Wait and watch: A trachoma surveillance strategy from Amhara region, Ethiopia
Source: PLoS Negl Trop Dis. 2024 Feb 22;18(2):e0011986. doi: 10.1371/journal.pntd.0011986 (PMC10914254; doi:10.1371/journal.pntd.0011986)

Supplemental Fig 1. Age-specific prevalence of TF among children ages 1 to 9 years, Amhara, Ethiopia, 2021.


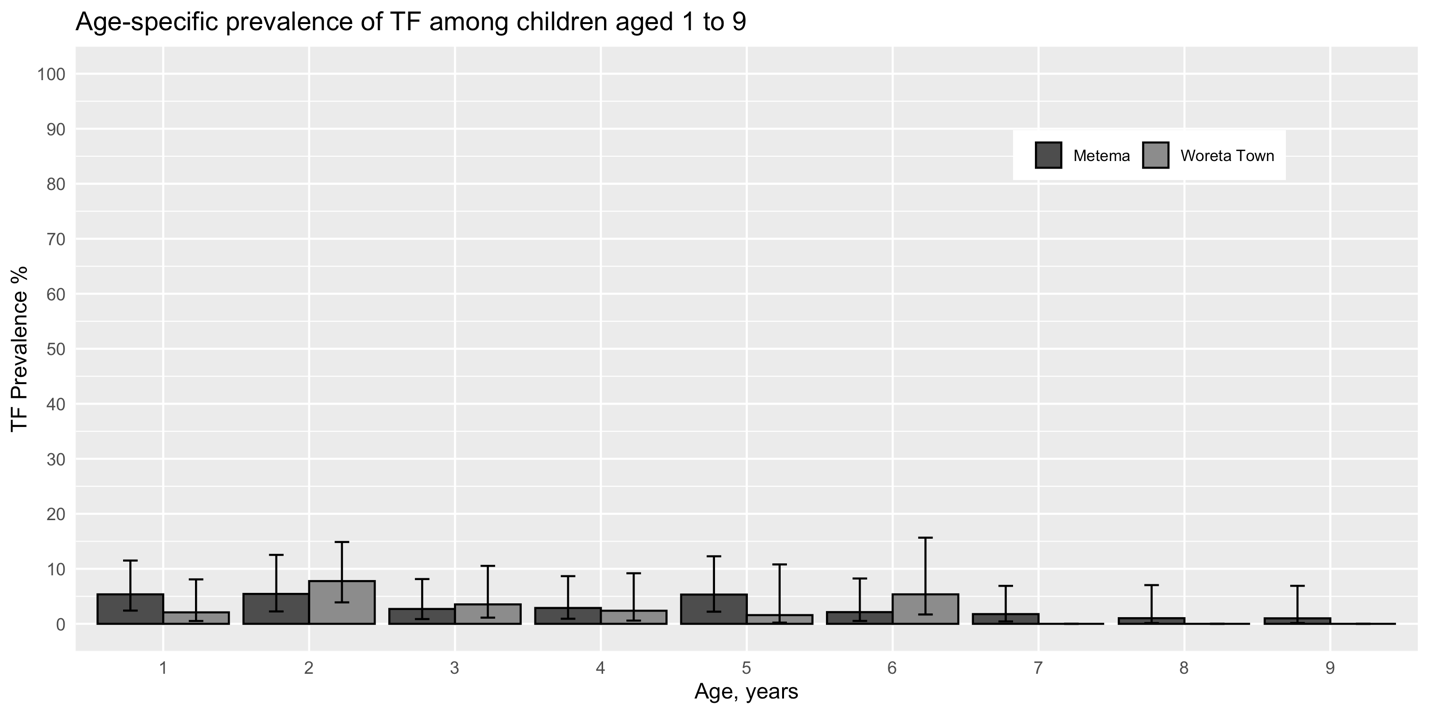

Supplement: S1 Fig — (DOCX) [file pntd.0011986.s002.docx]
